# Inhibitor of Hyaluronic Acid Synthesis 4-Methylumbelliferone as an Anti-Inflammatory Modulator of LPS-Mediated Astrocyte Responses

**DOI:** 10.3390/ijms21218203

**Published:** 2020-11-02

**Authors:** Dmitry V. Chistyakov, Arina I. Nikolskaya, Sergei V. Goriainov, Alina A. Astakhova, Marina G. Sergeeva

**Affiliations:** 1 Belozersky Institute of Physico-Chemical Biology, Lomonosov Moscow State University, 119992 Moscow, Russia; alina_astakhova@belozersky.msu.ru (A.A.A.); mg.sergeeva@gmail.com (M.G.S.); 2 SREC PFUR, Peoples’ Friendship University of Russia (RUDN University), 117198 Moscow, Russia; goryainovs@list.ru; 3 Faculty of Bioengineering and Bioinformatics, Moscow Lomonosov State University, 119234 Moscow, Russia; arnikol31@gmail.com

**Keywords:** astrocytes, 4-methylumbelliferone (4-MU), cyclooxygenase (COX), hyaluronic acid, interleukin 10 (IL-10), interleukin 6 (IL-6), neuroinflammation, oxylipins, toll-like receptors (TLRs)

## Abstract

Astrocytes are glial cells that play an important role in neuroinflammation. Astrocytes respond to many pro-inflammatory stimuli, including lipopolysaccharide (LPS), an agonist of Toll-like receptor 4 (TLR4). Regulatory specificities of inflammatory signaling pathways are still largely unknown due to the ectodermal origin of astrocytes. Recently, we have shown that hyaluronic acid (HA) may form part of astrocyte inflammatory responses. Therefore, we tested 4-methylumbelliferone (4-MU), a specific inhibitor of HA synthesis, as a possible regulator of LPS-mediated responses. Rat primary astrocytes were treated with LPS with and without 4-MU and gene expression levels of inflammatory (interleukins 1β, (IL-1β), 6, (IL-6), tumor necrosis factor alpha TNFα,) and resolution interleukin 10 (IL-10) markers were evaluated via real-time PCR and western blot. The release of cytokines and HA was determined by ELISA. Oxylipin profiles were measured by ultra-performance liquid chromatography-tandem mass spectrometry (UPLC-MS/MS) analysis. Our data show that 4-MU (i) has anti-inflammatory effects in the course of TLR4 activation, decreasing the cytokines level TNFα, IL-6 and IL-1β and increasing IL-10, (ii) downregulates prostaglandin synthesis but not via cyclooxygenases COX-1 and COX-2 pathways, (iii) modulates HA synthesis and decreases LPS-induced HA synthase mRNA expression (HAS-1, HAS-2) but does not have an influence on HAS-3, HYAL1 and HYAL2 mRNAs; (iv) the effects of 4-MU are predominantly revealed via JNK but not p38, ERK mitogen-activated protein kinases (MAPKs) or nuclear factor kappa-light-chain-enhancer of activated B cells (NF-kB) pathways. For the first time, it is shown that 4-MU possesses the useful potential to regulate an inflammatory astrocyte response.

## 1. Introduction

A significant amount of research is currently being devoted to the establishment of the mechanisms of inflammation and the search for possibilities of process regulation. It is known that inflammation accompanies all known neurological pathologies, including neurodegenerative diseases, post-ischemic neurodegeneration, traumatic, metabolic, toxic and neoplastic disturbances [1,2,3]. Although neuroinflammation is a form of the innate immune response, initiated by altered homeostasis within brain tissues, not only microglia, as cells of immune origin but other cells of the central nervous system (CNS) contribute to neuroinflammation through activation of Toll-like receptors (TLRs) [2,4,5,6]. Astrocytes are glial cells with homeostatic, metabolic and defensive functions and play an important role in the development of inflammatory responses in the brain [7]. Upon activation of TLR-mediated signaling pathways, astrocytes produce pro- and anti-inflammatory cytokines and polyunsaturated fatty acid derivatives, such as prostaglandins [7,8,9]. TLRs can be activated not only by various exogenous ligands, the most well-studied among them being LPS [10], which acts as a TLR4 agonist in astrocytes [11]. An array of endogenous molecules generated and released after cell activation by pro-inflammatory stimuli may be effective modulators of neuroinflammation processes [10]. Therefore, a detailed examination of TLR-mediated signaling pathways as an important element of neuroinflammation on cellular and molecular levels is crucial for new therapeutic targets discovery and the development of effective treatment strategies.

Glycosaminoglycan HA is present in the extracellular matrix (ECM) and exhibits diverse biological functions, including the response to tissue injury and inflammation [12,13,14]. Hyaluronan inhibition was suggested as a therapeutic strategy in inflammation, autoimmunity and cancer [15]. It is known that the ECM comprises approximately 20% of the central nervous system (CNS); HA is one of its major structural components that not only organizes heterogeneous populations of neurons and glial cells into highly structured functional units of the CNS but also acts as a regulator of some brain functions, playing a significant role in maintaining the homeostasis of the nervous tissue [16,17,18,19]. Several lines of evidence indicate that HA is linked to neuroinflammation in vitro and in vivo [20,21]. Recently, we have shown that HA added to cultured astrocytes influences on inflammatory markers: cytokines (Tumor necrosis factor alpha (TNFα), interleukin 6 (IL-6), interleukin 10 (IL-10)), enzymes (inducible nitric oxide synthase (iNOS), cyclooxygenases 2 (COX-2)) and oxylipins levels and therefore, HA modulates TLR4- and TLR3-signaling pathways [9]. Astrocytes themselves can synthesize HA and these cells are considered a major source of hyaluronan in the brain [22,23]. Therefore, the possibility of modulating the HA synthesis by astrocytes is a promising direction in the regulation of neuroinflammation.

One of the well-defined modulators of HA synthesis is 4-MU, a coumarin derivative. 4-MU is available as a spasmolytic, over-the-counter drug in several European countries (named “hymecromone”). It was shown that 4-MU is a specific inhibitor of the HA synthesis in multiple cell lines, including fibroblasts from various primary tissues [24,25,26,27], keratinocytes [28], melanoma and pancreatic cancer cells [29,30]. Moreover, the effect of 4-MU has been shown in certain in vivo experiments. 4-MU treatment prevented lung injury in mouse models of staphylococcal enterotoxin-mediated [31] and lipopolysaccharide-mediated acute lung injury [32]. 4-MU has also been shown to have protective effects on non-infectious inflammation, such as a model of renal ischemia-reperfusion injury [33], a murine atherosclerosis model [34], Graves’ orbitopathy [27] or models of hypertriglyceridemia and hyperglycemia, induced by a high-fat diet [35]. 4-MU has also been reported to improve the course of diseases in mouse models of autoimmune diseases, such as the collagen-induced arthritis model [24] and a brain autoimmunity model [20]. In view of these data, it is surprising that 4-MU has not been investigated in models of stimulating inflammation on cells of the nervous system. In the present study, we have filled this gap and shown that 4-MU can effectively ameliorate TLR-mediated signaling by modulating cytokines and oxylipins’ release. The effectiveness of 4-MU as an anti-inflammatory agent stimulated the following assessment of possible participants in signaling cascades—phosphorylation of mitogen-activated protein kinases (MAPK) p38, JNK, ERK, transcription factor NF-kB, expression of the enzymes of HA metabolism.

## 2. Results

### 2.1. LPS-Induced Release of Cytokines and Oxylipins Modulates by 4-MU

**Figure 1:**
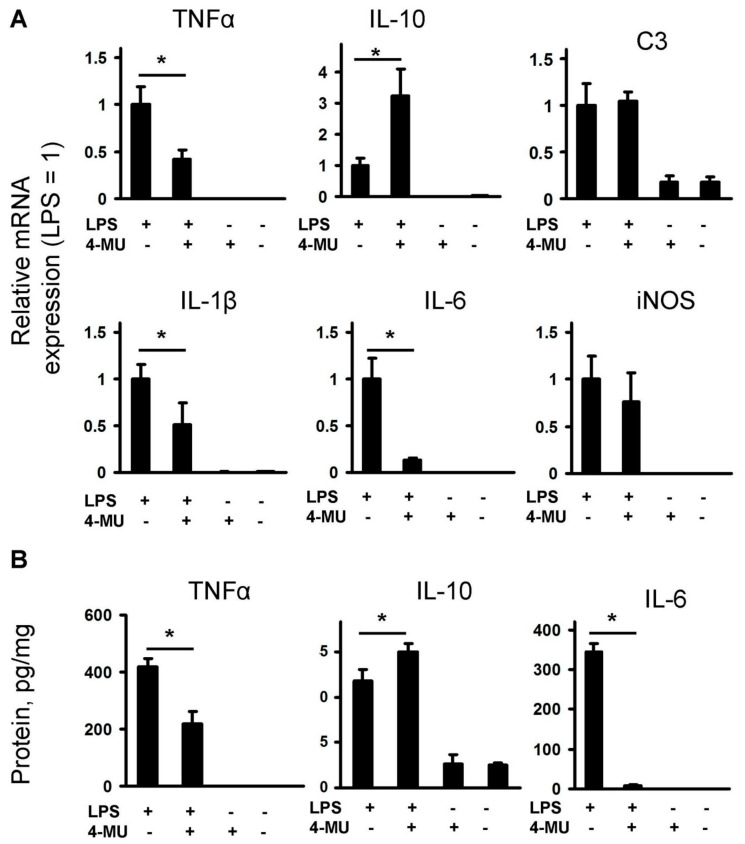
Effect of 4-MU on the inflammatory response. The primary rat astrocyte cultures were pretreated with 4-methylumbelliferone (4-MU, 400 μM) for 30 min and then stimulated with lipopolysaccharide (LPS) (100 ng/mL) for 4 h. (**A**): the mRNA levels of inflammatory markers (tumor necrosis factor alpha (TNFα), interleukin 10 (IL-10), complement component 3 (C3), interleukin 1 beta (IL-1β), interleukin 6 (IL-6) and inducible nitric oxide synthase (iNOS)) were determined by quantitative real-time PCR (qPCR). The values were normalized to β-actin mRNA levels. The results are expressed as fold-changes, relative to untreated cells. (**B**): the TNFα, IL-10 and IL-6 protein release were measured by ELISA in supernatant samples. The results are expressed as pg/mg. The values represent a mean ± SEM from three independent experiments. * *p* < 0.05, compared with the LPS-stimulated cells.

Previously, we have shown that the LPS-induced release of pro-inflammatory cytokines and oxylipins can be modulated by exogenously added HA [9]. As a next step, we tested how 4-MU can modulate the release of the pro-inflammatory substances. Astrocytes were pretreated with 4-MU for 30 min, then LPS (100 ng/mL) was added for 4 h and mRNA expression of pro-inflammatory markers (TNFα), IL-1β, C3, iNOS, IL-6 and anti-inflammatory marker IL-10 were estimated using the qPCR method (Figure 1A). 4-MU inhibits the LPS-induced mRNA expression of TNFα, IL-1β, IL-6, does not influence C3 and iNOS mRNA expression and potentiates IL-10 mRNA expression. The mRNA gene expression levels were correlated with protein levels for TNFα, IL-10 and IL-6 (Figure 1B). Therefore, 4-MU possesses not only anti-inflammatory activity via suppression of pro-inflammatory cytokines but even potentiates the expression of IL-10, a cytokine of inflammatory resolution [36].

**Figure 2:**
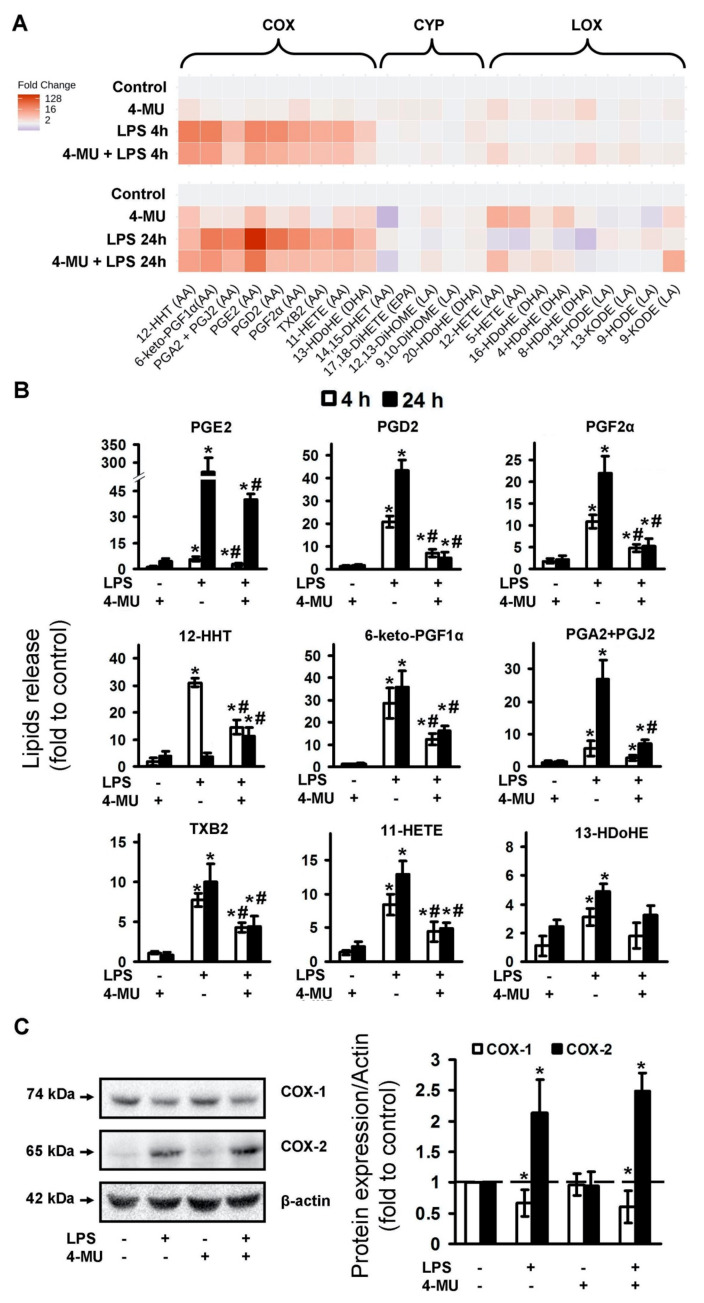
Effect of 4-methylumbelliferone (4-MU) on the oxylipins release and cyclooxygenases expression in the LPS-stimulated astrocytes. Primary rat astrocytes were pretreated for 30 min with 4-MU (400 μM) and then stimulated with lipopolysaccharide (LPS, 100 ng/mL) for 4 h or 24 h. Concentrations of oxylipins in supernatants were measured using ultra-performance liquid chromatography-tandem mass spectrometry (UPLC-MS/MS). (**A**) The heat map shows relative amounts of each lipid mediator compared to the control. The vertical axis indicates the stimuli, while the horizontal axis indicates the relative amount (log2) of each lipid mediator. Metabolites were divided into: Lipoxygenase (LOX), cyclooxygenase (COX) and cytochrome (CYP) pathways involved in their synthesis. (**B**) The bars show relative amounts of COX-derived lipid mediators. (**C**) COX-1 and COX-2 protein levels were evaluated by western blotting and normalized to the loading control β-actin. The example is representative for three independent experiments. Values represent the mean ± SEM from three independent experiments. * *p* < 0.05, compared with the unstimulated cells, # *p* < 0.05, compared with the LPS-stimulated cells.

It is known that, besides the release of pro- and anti-inflammatory cytokines, cellular responses to inflammatory stimuli are characterized by oxylipins synthesis [37]. Oxylipins are formed from polyunsaturated fatty acids (PUFAs) via lipoxygenase (LOX), cytochrome P450 (CYP), cyclooxygenase (COX) pathways or non-enzymatically [37,38,39]. These substances have tremendous effects on cellular responses, including pro- and anti-inflammatory actions via highly specific plasma membrane receptors, less specific nuclear receptors or other mechanisms [37,38]. Although many oxylipins are released in low concentrations, their effects can be summarized [39]. Therefore, only oxylipin profiles measured by mass-spectrometry methods can reveal this system’s features. We obtained oxylipin profiles for 4-MU modulations of oxylipin synthesis, measured in the extracellular medium for 4 and 24 h of LPS stimulations (Figure 2A). Data are presented as a heatmap, where the horizontal axis indicates the stimuli, while the vertical axis indicates the relative amount (log2) of each lipid mediator (Figure 2A).

LPS-treated cells demonstrated the most significant increase in the concentration of the COX-derived metabolites, that is, arachidonic acid (AA) metabolites 12-HHT, 6-keto-PGF1α, PGA2 + PGJ2, PGE2, PGD2, PGF2α, TXB2 and 11-HETE, as well as docosahexaenoic acid (DHA) metabolite 13-HDoHE (Figure 2A). 4-MU reduces the effects of LPS on the synthesis of these oxylipins, both at 4 and 24 h of stimulation (Figure 2B). Thus, the data allow us to conclude that the 4-MU has a clear anti-inflammatory effect on LPS-stimulated astrocytes, reducing the synthesis of pro-inflammatory cyclooxygenase-derived oxylipins.

The influence of 4-MU on oxylipin profiles stimulated with LPS (Figure 2B) suggests the involvement of COX-1 and COX-2, active participants in the synthesis of eicosanoids synthesis after inflammatory stimulation. Previously, we have shown that COXs are the main enzymes responsible for the LPS- induced synthesis of eicosanoids and other COX-derived oxylipins in astrocytes [9,40]. Therefore, we analyzed the modulation of LPS-induced COXs expression by 4-MU (Figure 2C). We found a significant decrease in COX-1 expression and an increase in COX-2 expression in the LPS-stimulated cells. Simultaneously, the tested concentration of 4-MU did not influence both LPS-induced COX-1 and COX-2 expression. This reveals that the effect of 4-MU on TLR4-mediated eicosanoid synthesis in astrocytes is not realized via the COX expression mechanism.

### 2.2. LPS Induces Expression of HA Synthase Enzymes and HA Release, Which Is Inhibited by 4-MU

**Figure 3:**
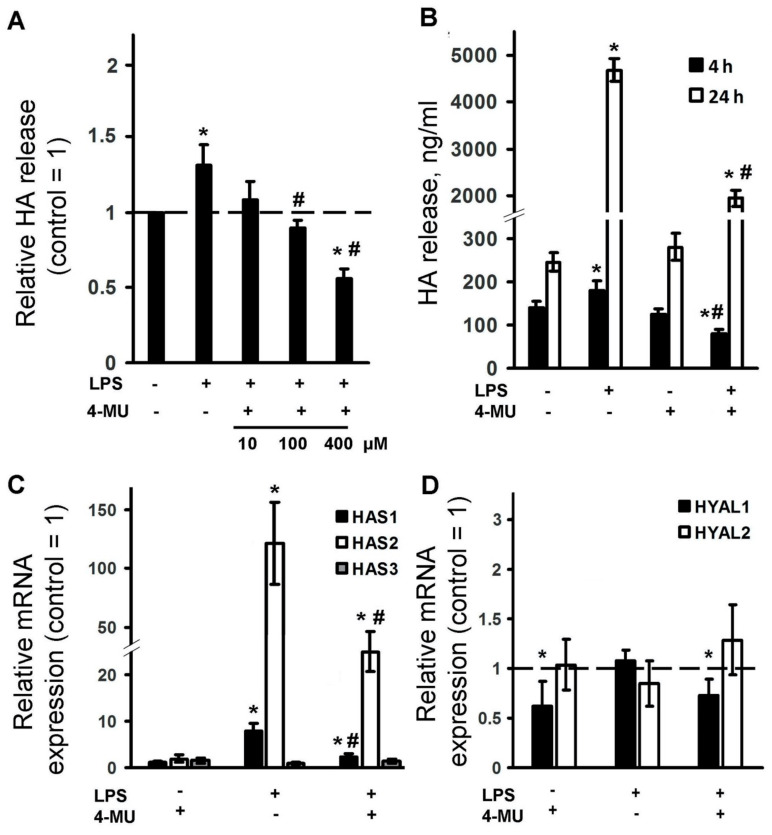
Modulation of LPS-induced hyaluronic acid (HA) release and mRNA expression levels of HA metabolizing enzymes, HA synthases (HAS1, HAS2, HAS3), hyaluronidases (HYAL1 and HYAL2) by 4-methylumbelliferone (4-MU). (**A**): the primary rat astrocyte cultures were pretreated with 4-MU, 10 μM, 100 μM or 400 μM) for 30 min and then stimulated with lipopolysaccharide (LPS, 100 ng/mL) for 4 h. The HA release is measured by ELISA in supernatant samples. The results are expressed as fold-changes, relative to untreated cells. (**B**–**D**): astrocytes were pretreated with 4-MU, 400 μM for 30 min and then stimulated with LPS (100 ng/mL) for 4 h (**B**–**D**) or 24 h (**B**). (**B**): the HA release was measured by ELISA in supernatant samples. The results are expressed as ng/mL. (**C**,**D**): the mRNA levels of HAS1, HAS2, HAS3, HYAL1 and HYAL2 were determined by quantitative real-time PCR (qPCR). The values are normalized to β-actin mRNA levels. The results are expressed as fold-changes, relative to untreated cells. * *p* < 0.05, compared with the naive cells, # *p* < 0.05, compared with the LPS-stimulated cells.

It is known that astrocytes can synthesize HA [22,23] and we have shown that HA can modulate LPS-mediated astrocyte responses [9]. Therefore, in the next set of experiments, we tested whether LPS can modulate HA release to extracellular medium and, if so, whether 4-MU inhibits this synthesis (Figure 3A,B). Indeed, LPS induced HA release after 4 h of application and the effect potentiated at 24 h (Figure 3B). The inhibitory effect of 4-MU depended on the concentration, it was observed even at 100 μM (Figure 3A) and the effect persisted for 24 h of incubation with LPS (Figure 3B). Note, the concentration of HA in unstimulated cells was not affected by 4-MU at either 4 or 24 h of incubation (Figure 3B). At the same time, 4-MU removed the LPS-stimulated increase in HA at both times of treatment.

The HA synthesis is carried out by three different HA synthase enzymes (HAS1, HAS2 and HAS3) [41]. HA’s breakdown is carried out by two isoforms of hyaluronidases, HYAL1 and HYAL2 [15]. We estimated expressions of these five enzymes after LPS stimulation and the sensitivity of these expressions for 4-MU exposition (Figure 3C,D). The effect of LPS treatment on HAS and HYAL isoforms mRNA levels was assessed by qPCR (Figure 3). After 4 h of LPS treatment, a moderate increase in HAS1 mRNA expression (5-fold) was observed (Figure 3C). A significant increase in HAS2 mRNA (120-fold) was observed (Figure 3C), while HAS3 (Figure 3C), HYAL1 and HYAL2 mRNAs (Figure 3D) were not modulated. 4-MU inhibits LPS-induced HAS1 and HAS2 mRNA expression (Figure 3C) and possesses a weak but significant inhibition of HYALs, being added along or in the presence of LPS (Figure 3D). Taken together, the data in Figure 3 suggest that the anti-inflammatory effect of 4-MU on cytokines and oxylipins, at least correlated with its effect on HA synthesis.

### 2.3. 4-MU Modulate TLR-Mediated JNK but Not p38, ERK MAPK and NF-kB Activities

**Figure 4:**
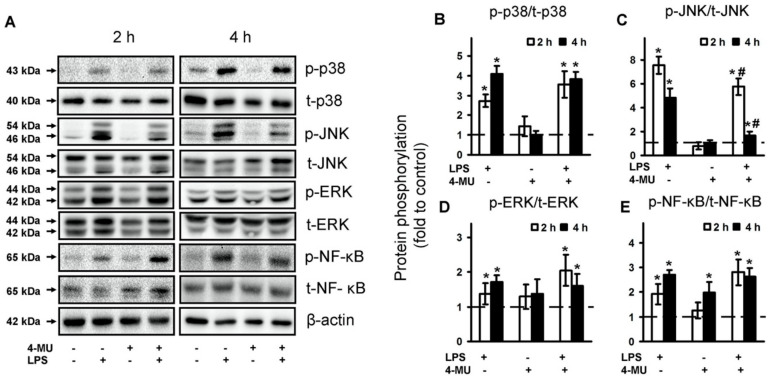
Comparison of NF-kB, p38, JNK and ERK1/2 MAPK activity in the LPS-stimulated astrocytes, treated with 4-MU. Astrocytes were pretreated for 30 min with 4-methylumbelliferone (4-MU, 400 μM) and subsequently kept for 4 h without any additional stimulation or with lipopolysaccharide (LPS, 100 ng/mL). p38, p-p38, pJNK, JNK, pERK1/2, ERK1/2 mitogen-activated protein kinases (MAPKs), nuclear factor kappa-light-chain-enhancer of activated B cells (p-NF-KB and NF-kB) protein levels were evaluated by western blotting at indicated time (given in h) and normalized to the loading control β-actin. (**A**) Representative Western blots demonstrating phospho-p38 (p-p38), phospho-JNK (p-JNK), phospho-ERK1/2, phospho-NF-kB and total p38 (p38), total JNK (JNK), total ERK1/2 and total NF-kB protein levels. The example is representative for three independent experiments. (**B**–**E**) Results are expressed as fold-changes, relative to untreated control astrocytes. Values represent mean ± SEM from three independent experiments. * *p* < 0.05, compared with the unstimulated cells, # *p* < 0.05 compared with the LPS-stimulated cells.

Mitogen-activated protein kinases (MAPKs) are components of TLR-mediated signaling and their roles in astrocytes are intensively investigated [11,42,43,44]. The transcription factor NF-kB is involved in the TLR-mediating signaling process in all cells [45]. To evaluate the intracellular mechanisms of 4-MU action, we investigated its effect on the phosphorylation of NF-kB, p38 MAPK, Jun N-terminal kinase (JNK) MAPK and p44/42 MAPK (ERK) (Figure 4). As in the previous sets of experiments, astrocytes were pretreated for 30 min with 4-MU then exposed for 4 h with and without LPS (100 ng/mL). p38, p-p38, pJNK, JNK, pERK1/2, ERK1/2, p-NF-kB and NF-kB protein levels were evaluated by western blotting (Figure 4A). To avoid the time-dependence of phosphorylation, we also tested LPS stimulation over a 2 h period (Figure 4). Indeed, LPS induced phosphorylation of p38 MAPK and its effect increased over time (Figure 4B). The activation of TLR4 signaling pathways induced the upregulation of all measured protein for 4 h with peaks at 2 h for JNK (Figure 4B–D). The LPS-mediated increase in phosphorylation of JNK was stronger at 2 h than at 4 h (Figure 4C). Less significant was the increase in phosphorylation levels for ERK (Figure 4D) and NF-kB (Figure 4E). 4-MU being added without stimulation did not have an influence on MAPKs phosphorylation levels (Figure 4A–D). We saw an increase in the phosphorylation level of NF-kB, pretreated with 4-MU for 4 h (Figure 4E), that could manifest in the complexity of 4-MU effects. The notable decrease in MAPK phosphorylation levels for cells treated by 4-MU with subsequent stimulation by LPS was significant only for JNK, the effects presented both after 2 and 4 h of stimulation (Figure 4C). The data suggest that certain 4-MU effects in LPS-stimulated astrocytes may be attributed to JNK-mediated processes.

## 3. Discussion

Our data demonstrated that treatment with the HA synthesis inhibitor 4-MU may be effective in relation to LPS-mediated inflammatory responses in astrocytes. Exposure of astrocytes to 4-MU led to a decreased synthesis of pro-inflammatory cytokines (TNFα, IL-6) and prostaglandins. At the same time, the treatment led to an increase in IL-10 synthesis, which is considered to be a cytokine of resolution. These effects were characterized by the reduced production of HA, a decreased LPS-mediated expression of HAS1 and HAS3 and decreased activation of the JNK MAPK. Taken together, the present study reveals 4-MU to be a potentially promising substance with anti-inflammatory and pro-resolution activities in brain inflammatory processes.

It was shown that 4-MU reduced the LPS-stimulated up-regulation of inflammatory cytokines (IL-1, IL-6, IL-8, TNF-α) in the corneal fibroblasts [26]. The drug decreased LPS-induced cytokine production (IL-1, IL-6 and TNF-α) in spleen cell cultures [32]. Our data are consistent with these results. Such consistency between various cell types is not obvious because LPS-mediated signaling in astrocytes has many specific regulatory features [46,47]. It is possible to detect a similarity between the involvement of 4-MU in LPS-induced inflammatory responses in various cell types. Our data have also indicated a positive influence of 4-MU on IL-10 induction. This fact allows us to discuss the action of 4-MU not only as an anti-inflammatory substance but also in terms of acting as a stimulant with regard to the resolution processes.

The suppressive effect of 4-MU on prostaglandin synthesis also reveals its anti-inflammatory features. To our knowledge, the connection between 4-MU and oxylipin synthesis has not been estimated before. There are data concerning the regulation of HA-synthesis by prostaglandins and even evidence of cyclooxygenase-2/PGE2 playing a central role in the regulation of HA-synthesis during atherogenesis [48]. Although it was shown that human vascular smooth muscle cells upregulate HAS1 and HAS2 in response to the activation of prostaglandin receptors (subtypes IP and EP2) [48], the feedback mechanism of the influence of HA synthesis inhibitors on prostaglandin synthesis has not yet received attention. Our data pose an interesting question concerning the mechanisms of the drug action, as we did not see any modification of COX-2 or COX-1 expression, however, 4-MU induced a significant inhibition of the cyclooxygenase pathway of oxylipin synthesis. Understanding the mechanism of the process requires further study.

Firstly, 4-MU is used as a low toxicity, high potency inhibitor of HA biosynthesis [15]. Our data with LPS-induced HA synthesis reveal a similarity between the astrocytes and other cell types. Indeed, the effect of 4-MU on HA synthesis was first reported in human fibroblasts and has since been confirmed in different cell types, including many tumors [15,49]. LPS or other pro-inflammatory stimuli-induced expressions of HASs that are accompanied by HA synthesis [26]. The inhibition of HA synthesis by 4-MU is accompanied by a decrease in mRNA HASs expression in many cell types [26,50]. In our work, the expression of the three types of HAS was detected in astrocytes and LPS stimulation and caused an up-regulation of HAS1 and HAS2 expression in astrocytes, which is consistent with previously published data in corneal fibroblasts [26]. The same concentration-dependence effect of 4-MU on HA release was previously shown for rat epidermal keratinocyte (REK) cultures [28]. In our experiments, 4-MU inhibits HAS1 and especially HAS2, which is considered to be involved in the synthesis of high molecular weight-HA (HMW-HA) [51]. This apparent negative regulatory loop is inconsistent with data previously obtained for corneal fibroblasts, where both 4-HU and HMW-HA decrease LPS-induced pro-inflammatory cytokines release [26]. Recently we have shown that the short-term application of HMW, together with LPS, increased TNFα release in astrocytes [9]. The possible explanation for these discrepancies may be the measurable time of cellular responses; the study with fibroblasts used 48 h of exposition with LPS and substances, whereas we used 4 h. Previously we have shown that long-term HMW-HA exposition does not influence LPS-induced 48 h astrocytes’ responses [9]. Similar results indicating that the 4-MU anti-inflammatory effect was not correlated with its inhibition with HA synthesis were obtained previously for chondrocytes [52]. Therefore, although 4-MU decreases LPS-induced HAS’s expression, the question concerning 4-MU and HMW-HA relationships in astrocytes remains.

Concerning mechanisms of cell signaling, we have shown that the effect of 4-MU is specific and decreases JNK phosphorylation without attenuation of NF-kB, p38 MAPK or ERK. The data are in accordance with the previously published effect of 4-MU on IL-1-stimulated, human or bovine chondrocytes, where no changes in NF-kB, p38 and ERK MAPK were obtained (JNK was not measured) [52]. Many studies involving the measurement of protein phosphorylation have been performed on cancer cell lines. The involvement of 4-MU in the inhibition of ERK phosphorylation was shown in the case of malignant pleural mesothelioma (MPM) cells [53] and esophageal squamous cell carcinoma cell lines [54,55]. 4-MU inhibited NF-kB reporter activity and decreased phospho-IKB levels in prostate cancer cells [56]. 4-MU led to a significant increase in p-p38 in the K562 chronic myelogenous leukemia cells [57]. Note that the addition of 4-MU induced caspase-dependent apoptosis, characterized by decreased HA production and increased phosphorylation of p38 [57]. Thus, it seems that the mechanisms of 4-MU action differ between cancer and normal cells. At the same time, the involvement of JNK MAPK in the anti-inflammatory effects of 4-MU has not been previously shown.

The molecular mechanisms of 4-MU anti-inflammatory action and interconnection with HA synthesis is still not obvious. There is a possibility of 4-MU action with multiple mechanisms [52]. Indeed, HA is normally synthesized by HAS1, HAS2 and HAS3 at the plasma membrane, using cytosolic UDP-glucuronic acid (UDP-GlcUA) and UDP-*N*-acetyl-glucosamine (UDP-GlcNAc) substrates [41,58]. Both substrates are generated by the transfer of a UDP-residue to glucuronic acid or *N*-acetylglucosamine by UDP-glucuronosyltransferase (UGT) and the availability of the substrate’s controls HA synthesis [15,50,59]. UDP-GlcNAc is also the substrate for O-GlcNAc transferase, which is central to the control of many cytosolic pathways and there is a hypothesis that the hyaluronan metabolism system works like a rheostat for controlling an acceptable normal range of cytosolic UDP-GlcNAc concentrations in order to maintain normal cell functions [41]. Within this system, 4-MU covalently binds through its hydroxyl group at position four to glucuronic acid via the UGT, forming 4-MU-glucuronic acid, which is not a substrate of HASs. 4-MU thereby reduces the UDP-GlcUA content inside the cells and inhibits HA synthesis [41]. This effect of 4-MU as a substrate catcher was shown [15,50]. Besides this, the 4-MU treatment decreased mRNA HASs expression [26,50] and reduced expression of mRNA for UDP-glucose pyrophosphorylase and dehydrogenase, which are critical enzymes for the synthesis of the HA precursors [60]. We have only found one work concerning the interaction of astrocytes and 4-MU. The substance was used as a marker of UDP-glucuronosyltransferase activity, measured in cell lysates [61]. Glucuronidation activity and the expression of UGT1A6 isoform of UDP-glucuronosyltransferase increased after the astrocytes’ exposure to LPS [61]. Therefore, multiple mechanisms of 4-MU action in astrocytes should be taken into consideration.

A very interesting feature of HA’s regulation in astrocytes is a difference in its regulation in naive and LPS-stimulated cells. Without LPS, the HA concentration depended only on the cell density and cultivation time; 4-MU did not interfere with this process despite its effectiveness in inhibiting LPS-mediated HA release. This indicates the possibility of controlling extracellular HA by several regulatory mechanisms. Recently it was shown that astrocytes constitutively express TSG-6 (Tumor necrosis factor (TNF)-stimulated gene-6) in the adult brain and spinal cord and the expression of this extracellular protein was up-regulated after injury and presented within the HA-rich glial scar [62]. TSG-6 belongs to the family of hyaladherins, binds with many targets and has anti-inflammatory and tissue-protective properties in various tested cellular models [63]. Although its expression usually appears at a more prolonged time than in the present study [62,64], it is attractive to suppose the involvement of the protein in HA regulation in astrocytes. In any case, the interrelation between TLR-mediated signaling in astrocytes and their extracellular matrix appears to be more complex and time-dependent than previously thought.

In summary, 4-MU provides for the selective inhibition of anti-inflammatory and pro-resolution activity in astrocytes. 4-MU is also a useful tool in blocking the production of HA. Although the inhibition of HA biosynthesis occurs, our study demonstrates that 4-MU may exert effects independent of its effects on HA. The fact that 4-MU is a drug used in clinical practice creates additional benefits for a more detailed study of its effect on the CNS in various pathologies with an inflammatory component.

## 4. Materials and Methods

### 4.1. Reagents

Lipopolysaccharide (cat.no. L2630) and 4-methylumbelliferone (cat.no. M1381) were from (Sigma-Aldrich, St. Louis, MO, USA), streptomycin–penicillin (cat.no. A063), trypsin (cat.no. P037), EDTA, fetal bovine serum (cat.no BS-110/500) were from PanEco (Moscow, Russia). Culture medium Dulbecco’s Modified Eagle Medium (DMEM) (cat.no. 21885-025) (Gibco, Thermo Fisher Scientific, Waltham, MA, USA). Antibodies against COX-2 (cat.no. 12282), COX-1 (cat.no. 4841, Danvers, MA, USA), p38 MAPK (cat.no 9212), phospho-p38 MAPK (Thr180/Tyr182, cat.no. 9211), SAPK/JNK (cat.no. 9252), phospho-SAPK/JNK (Thr183/Tyr185, cat.no. 4668), p44/42 MAPK (Erk1/2, cat.no. 9102), phospho-p44/42 MAPK (Erk1/2, Thr202/Tyr204, cat.no. 9106), NF-κB p65 (cat.no. 8242), phospho-NF-κB p65 (Ser536, cat.no. 3033) were from (Cell Signaling Technology, Danvers, MA, USA) and β-actin (Cell Signaling Technology, Danvers, MA, USA), secondary horseradish peroxidase conjugated antibodies (anti-rabbit, anti-mouse and anti-goat) (Cell Signaling Technology, Danvers, MA, USA), western blotting substrate ECL (Thermo Fisher Scientific, cat.no 32209, Waltham, MA, USA) were used. ELISA kits for TNFα (cat.no. 558535), IL-10 (cat.no. 555134) and IL-6 (cat.no. 550319) from BD Biosciences, San Diego, USA, San Diego, CA, USA, Hyaluronic acid (cat.no. DHYAL0) from R & D Systems, USA, Minneapolis, MN were also used. The oxylipins standards were as follows: 6-keto PGF1α-d4 (cat.no. 315210), TXB2-d4 (cat.no. 319030), PGF2α-d4 (cat.no. 316010), PGE2-d4 (cat.no. 314010), PGD2-d4 (cat.no. 312010), 5(S)-HETE-d8 (cat.no. 334230), 12(S)-HETE-d8 (cat.no. 334570), 15(S)-HETE-d8 (cat.no. 334720) (Cayman Chemical, Ann Arbor, MI, USA). Oasis^®^ PRIME HLB cartridges (60 mg, 3cc, cat.no. 186008056) were obtained from Waters, Eschborn, Germany.

### 4.2. Primary Cell Culture

The cells were obtained from one- or two-day-old pups of Wistar rats. All of the experimental procedures were performed according to the guidelines in the European Convention for the Protection of Vertebrate Animals used for Experimental and Other Scientific Purposes, and were approved by the Bioethics Committee (Protocol 2/13 from 8 April 2013) of The Department of Biology at Moscow State University. The cultures of primary rat astrocytes were obtained from newborn rats of both sexes, as previously reported [46]. In brief, the brains from decapitated pups were triturated against nylon meshes with the pores of 250 and 136 μm, in a consecutive order. The dissociated cells were plated into 75 cm^2^ culture flasks at a density of 6 × 10^^5^ cells per mL. The cells were subsequently cultured in DMEM (1 g/L D-glucose, 10% bovine fetal serum (FBS), 50 units/mL streptomycin, 50 μg/mL penicillin) at 37 °C, with 10% CO2. After five days of cultivation in DMEM, the culture medium was replaced with a fresh medium and the flasks were placed on a shaker at 200 rpm for 4 h to dissociate the microglial cells. The microglia-containing medium was discarded and the astrocytes-enriched cultures were further grown for the following four days and the medium was replaced every two days. Subsequently, the cells were plated into six-well plates and were maintained for two days in DMEM. After this, the medium was replaced by the medium of the same composition and the cells were used for the experiments. The effect of treatments on cell viability MTT assay was used to determine changes in viability following exposure of confluent primary astrocyte cultures to 4-MU and other pre- and co-treatments. No loss of viability occurred in astrocyte cultures (data not shown).

### 4.3. Measurement of the Relative RNA Expression Level

We estimated the expression of genes in rat astrocyte cultures—TNFα (Tumor necrosis factor alpha), IL-10 (Interleukin 10), C3 (complement component 3), IL-1β (Interleukin 1 beta), IL-6 (Interleukin 6) and iNOS (inducible nitric oxide synthase), HAS1 (Hyaluronan synthase 1), HAS2 (Hyaluronan synthase 2), HAS3 (Hyaluronan synthase 3), HYAL1 (Hyaluronidase-1), HYAL2 (Hyaluronidase-2).

Total mRNA was isolated using the GeneJET RNA Purification Kit (Thermo Scientific, Waltham, MA, USA). The concentration of RNA was measured using an Implen NanoPhotometer C. cDNA was generated according to the manufacturer’s instructions using the MMLV RT kit (Evrogen, Moscow, Russia) with oligo-(dT)-primers. Real-time PCR was performed using the 5 × PCR-HS-SYBR mix (Evrogen, Moscow, Russia) and the DTlite 4 amplificator (DNATechnology, Moscow, Russia). The β-actin gene was used as a constitutive gene for normalization. The level of normalized gene expression in control cells or in stimulated cells (specified directly in the text) was taken as one. The sequences of PCR primers used in this study are presented in Table 1.

### 4.4. Western Blot Analysis

Western blot analysis was performed as described earlier [44]. In brief, astrocytes were lysed in a modified radio immunoprecipitation assay (RIPA) buffer (50 mM Tris, pH 7.4, 1% NP-40 Sigma Chemicals (St. Louis, MO, USA), 0.25% Na-deoxycholate, 150 mM NaCl, 1 mM EDTA, 1 mM Na_3_VO_4_, 1 mM NaF) and protease/phosphatase inhibitor cocktail (Roche Molecular Biochemicals, Mannheim, Germany). The protein concentration was determined by the standard Bradford assay. Samples containing 20 μg of protein in a conventional Laemmli buffer were loaded on each lane of a 10% sodium dodecyl sulfate-polyacrylamide gel and subjected to a standard SDS-PAGE. After electrophoresis, the proteins were transferred onto the nitrocellulose membrane with 0.2 μm pores. The membranes were blocked in a 10% Rotiblock (Roth, Nürnberg, Germany) solution for 1 h and subsequently subjected to a Phosphate-Buffered Saline with Tween 20 0.05%, with a respective primary antibody (1:1000) at 4 °C overnight. Secondary species-specific antibodies were applied at the concentration of 1:10,000 for 1 h at room temperature. The conjugates were visualized using the Pierce ECL Plus Western Blotting Substrate (Thermo Scientific, Waltham, MA, USA). First, phosphoproteins were detected, then the western blot membranes were re-probed (Mild stripping buffer (Abcam, Cambridge, UK) was used) and re-analyzed for total protein and then tested for β-actin. Densitometry was carried out on three different experiments. The band intensity was measured using a ChemiDoc™ XRS+ gel documentation system (Bio-Rad, Hercules, CA, USA) and normalized to the intensity of the respective bands obtained for β-actin.

### 4.5. UPLC-MS/MS Conditions and Sample Preparation

After the cell experiments, the supernatant was collected and stored at −80 °C for further analysis. The cell-free culture media were taken for the solid-phase lipid extraction (Oasis^®^PRIME HLB cartridge (60 mg, 3 cc)) as described previously [8]. Oxylipins were analyzed using 8040 series ultra-performance liquid chromatography-tandem mass spectrometer (Shimadzu, Japan) in multiple-reaction monitoring mode at a unit mass resolution for both the precursor and product ions. The molecular ions were fragmentized by collision-induced dissociation in the gas phase and analyzed by tandem (MS/MS) mass spectrometry. The selected lipids were identified and quantified by comparing their mass-spectrometric and chromatographic data with those obtained for the corresponding oxylipins standards (see M and M Section 4.1.) using Lipid Mediator Version 2 software (Shimadzu, Japan). The concentration of lipids was normalized to the total protein and was expressed as pg/mg. The total protein was determined by the Bradford assay.

### 4.6. Determination of TNFα, IL-10, IL-6 and Hyaluronic Acid by Enzyme-Linked Immunoassay

After the experiments, supernatants were collected and stored at −70 °C for further analysis. The levels of the released hyaluronan (R & D Systems) TNFα, IL-10 and IL-6 (BD OptEIA, BD Biosciences, San Jose, CA, USA) were determined using an enzyme-linked immunoassay commercial kits and Synergy H4 plate reader (BioTek, Winooski, VT, USA), following the manufacturer’s instructions.

### 4.7. Experimental Data Analysis and Statistics

The data are expressed as mean ±SEM. The normality of data sets was assessed using the Shapiro-Wilk test. The data were subjected to a one-way ANOVA, followed by Bonferroni’s post hoc test, in order to determine the statistical significance. *p* < 0.05 was considered statistically significant. All of the experiments were repeated at least three times.

## Figures and Tables

**Table 1:** DNA Sequences of the Primers used for RT-PCR.

Gene	Forward	Reverse
Tumor necrosis factor alpha *TNFα*	CAAGGAGGAGAAGTTCCCAA	TGATCTGAGTGTGAGGGTCTG
Interleukin 10 *IL-10*	CCCAGAAATCAAGGAGCATTTG	TCATTCTTCACCTGCTCCAC
Complement component 3 *C3*	AAGCCCAACACCAGCTACATC	ACTTCTGATCCTGGCATTCTTCT
Interleukin 1 beta *IL-1β*	CACCTCTCAAGCAGAGCACAG	GGGTTCCATGGTGAAGTCAAC
Interleukin 6 *IL-6*	CTGGTCTTCTGGAGTTCCGT	TGGTCTTGGTCCTTAGCCAC
Inducible nitric oxide synthase *iNOS*	CCACAATAGTACAATACTACTTGG	ACGAGGTGTTCAGCGTGCTCCACG
Hyaluronan synthase 1 *has1*	AGGTGCTGTTGGAGGAGATGTGA	AAGCTCGCTCCACATTGAAGGCTA
Hyaluronan synthase 2 *has2*	CCAATGCAGTTTCGGTGATG	ACTTGGACCGAGCCGTGTAT
Hyaluronan synthase 3 *has3*	CCTCATCGCCACAGTCATACAA	CCACCAGCTGCACCGTTAGT
Hyaluronidase-1 *hyal1*	TCGGACCCTTTATCCTGAAC	TTCTTACACCACTCTCCACTC
Hyaluronidase-2 *hyal2*	TCAGTGTACGCTTCAAGTATGGA	GACTGAGGTGCAAGAAGGTACTG
*β-actin*	AGATGACCCAGATCATGTTTGAG	GGCATACAGGGACAACACAG

## Abbreviations

AA	Arachidonic acid
COX	Cyclooxygenase
DHA	Docosahexaenoic acid
HA	hyaluronic acid
IL-10	interleukin-10
IL-6	interleukin 6
iNOS	inducible nitric oxide synthase
LOX	Lipoxygenase
LPS	lipopolysaccharide
PG	Prostaglandin
PUFAs	Polyunsaturated fatty acids
TLR	toll-like receptor
TNFα	tumor necrosis factor alpha
UPLC-MS/MS	Ultra-performance liquid chromatography-tandem mass spectrometry
